# Genetic Screening for *Arabidopsis* Mutants Defective in *STA1* Regulation under Thermal Stress Implicates the Existence of Regulators of Its Specific Expression, and the Genetic Interactions in the Stress Signaling Pathways

**DOI:** 10.3389/fpls.2016.00618

**Published:** 2016-05-10

**Authors:** Si-in Yu, Jin-Hee Han, Chanvotey Chhoeun, Byeong-ha Lee

**Affiliations:** Department of Life Science, Sogang UniversitySeoul, South Korea

**Keywords:** cold stress, heat stress, thermal stress, STA1, STABILIZED1, luminescence screening, housekeeping gene

## Abstract

To cope with environmental stresses, plants have developed various stress tolerance mechanisms that involve the induction of many stress responsive genes through stress-specific and common signaling pathways. Stress-specific/common transcription factors, rather than general basal factors, were considered important in this stress tolerance. The *Arabidopsis STABILIZED1* (*STA1*) gene encodes a putative pre-mRNA splicing factor that is similar to the human U5 snRNP-associated 102-kDa protein and the yeast pre-mRNA splicing factors, PRP1p and Prp6p. As pre-mRNA splicing is a necessary process for proper gene expression in eukaryotes, *STA1* is expected to be constantly functional in all conditions. Interestingly, *STA1* expression is induced by temperature stresses, and *STA1* recessive mutation (*sta1-1*) resulted in temperature stress-specific hypersensitivity. This suggests *STA1*’s stress specific function in addition to its presumed “housekeeping” role. In order to establish the genetic system to understand the regulation of *STA1* expression in temperature stresses, we generated a bioluminescent *Arabidopsis* plant harboring the *STA1* promoter fused to the firefly luciferase coding sequence (*STA1p-LUC*). Through genetic analysis, the bioluminescent *Arabidopsis* homozygous for one-copy *STA1p-LUC* was isolated and characterized. In this *STA1p-LUC* line, the expression patterns of *STA1p-LUC* were similar to those of the endogenous *STA1* gene under cold and heat stresses. The *STA1p-LUC* line was then chemically mutagenized and screened to isolate the genetic loci of *STA1* regulators under cold or heat stresses. Mutants with altered *STA1p-LUC* luminescence were identified and further confirmed through luminescence imaging in the next generation and analysis of endogenous *STA1* expression. The categorization of *STA1p-LUC* deregulated mutants implicated the existence of cold or heat stress-specific as well as common genetic regulators for *STA1* expression. Interestingly, some mutants showed opposite-directional deregulation of *STA1* expression depending on the type of thermal stress, suggesting that the loci may represent important switch factors which determine the direction of signaling pathways for *STA1* expression in response to temperature.

## Introduction

To cope with environmental extremes, plants have evolved a variety of strict controls on gene regulation to induce stress tolerance genes (Ingram and Bartels, 1996; Zhu, 2002; Chinnusamy et al., 2007; von Koskull-Döring et al., 2007). For example, most temperate plants develop freezing tolerance after prior-exposure to non-freezing temperatures; this phenomenon is known as cold acclimation (Guy, 1990). During cold acclimation, many physiological and molecular changes take place including the remodeling of cell/tissue structures, the re-programming of metabolism, and most importantly, changes in gene expression (Guy, 1990; Chinnusamy et al., 2007). The regulation of gene expression changes by cold stress is controlled by multiple transcriptional and translational programs (Chinnusamy et al., 2007; Zhu et al., 2007; Knight and Knight, 2012; Zhao et al., 2015). So far, our knowledge on regulation of cold-induced gene expression includes *ICE1* and *CBF* transcription factors which bind the promoters of its target genes and induce the cold-responsive genes, respectively (Chinnusamy et al., 2007; Knight and Knight, 2012; Zhao et al., 2015). This transcriptional program is one of the most well-known regulatory programs in cold signal transduction for cold-induced gene expression.

Similarly, heat stress signaling for tolerance acquisition is mediated by *MBF1c* (Multiprotein Bridging Factor 1c), *HSFs* (Heat Shock Factors), and some other transcription factors (Busch et al., 2005; Kotak et al., 2007; von Koskull-Döring et al., 2007; Saidi et al., 2011; Mittler et al., 2012). The heat signaling pathways through transcription factors are not linear, but rather complex and interconnected. These transcription regulators bind either directly or indirectly to promoter *cis*-elements of target genes to elicit heat stress response and tolerance (Kotak et al., 2007; Saidi et al., 2011; Mittler et al., 2012).

In fact, it is well known that stress signaling pathways for stress tolerance gene transcription are interconnected (Xiong et al., 2002; Shinozaki et al., 2003; Yamaguchi-Shinozaki and Shinozaki, 2006). Thus, identification of common and specific signaling factors would help in understanding the complex controls of stress tolerance gene regulation. Indeed, many shared signaling components have been isolated in cold and osmotic stresses (Yamaguchi-Shinozaki and Shinozaki, 2006). Recently, it was reported that some sets of metabolites were altered under cold and heat stresses, commonly as well as specifically (Kaplan et al., 2004). Despite this, cold- and heat-shared/specific signaling pathways and their target gene expressions have not been intensively investigated.

The *Arabidopsis* nuclear *STABILIZED1* (*STA1*) gene encodes a putative pre-mRNA splicing factor that is similar to the human U5 snRNP-associated 102-kDa protein (PRPF6) and the pre-mRNA splicing factors, PRP1p and Prp6p of fission and budding yeast; respectively. The *STA1* gene is expressed throughout the whole plant body and the STA1 protein is localized in the nuclei (Lee et al., 2006; Dou et al., 2013). *STA1* was shown to be involved in RNA stability, pre-mRNA and primary microRNA processing (Lee et al., 2006; Ben Chaabane et al., 2013). Recently, *STA1* was reported to play a role in RNA-directed DNA methylation (Dou et al., 2013). Complete knock-out of this gene has resulted in embryo lethal (Lee et al., 2006), and there is no paralog of *STA1* gene in the *Arabidopsis* genome (i.e., one copy gene in *Arabidopsis*). Taken together, these results implicated the basal and constant roles of the *PRP6* splicing factor homolog *STA1* in crucial and basic cellular processes (i.e., RNA metabolism and epigenetic regulations). Thus, it appears that *STA1* belongs to a group of so-called “housekeeping” genes that play basal roles in cellular systems (Butte et al., 2001; She et al., 2009).

Interestingly, the *sta1-1* mutant, a weak mutant allele of *STA1* gene, showed stress hypersensitive phenotypes mainly in temperature stresses (Lee et al., 2006). This raised an interesting question: how does the defect in this apparent “housekeeping” gene show stress-specific hypersensitivity? In this regard, induction of *STA1* mainly by low or high temperature stresses may be related to stress-specific function. Therefore, understanding the mechanisms of *STA1* induction by stresses is important to elucidate the specific functions of *STA1*. Also, studies of *STA1* regulation would provide clues about the common and specific signaling pathways for cold and heat stresses.

In order to understand the gene regulation and functional specificity of *STA1*, we developed an *Arabidopsis STA1* gene expression monitoring system by generating bioluminescent *Arabidopsis* plant harboring the transgene of *STA1* promoter-driven luciferase (*STA1p-LUC*). We confirmed that one copy of the *STA1p-LUC* transgene was inserted into the *Arabidopsis* genome and that this insertion did not interfere with neighboring gene expression. We also generated a *STA1p-LUC* derived mutant pool through chemical mutagenesis and isolated *STA1p-LUC* deregulated mutants showing similar alterations in both *STA1p-LUC* expression and endogenous *STA1* expression under temperature stress. Categorization of *STA1p-LUC* deregulated mutants indicated the existence of heat or cold stress-specific regulators in addition to common genetic regulators for *STA1* gene expression.

## Materials and Methods

### Plant Growth and Stress Treatment

*Arabidopsis* seeds were surface-sterilized with bleach solution (commercial bleach solution with 0.01% Tween 20) for 5 min and rinsed five times with sterile water. The seeds were planted on Murashige and Skoog (MS) medium plates that were made with full strength MS salts (Caisson Laboratories, US), 0.3% gelite (Duchefa, Netherland) and 2% sucrose (pH = 5.8). For selection plates, hygromycin was added to a final concentration of 25 mg/L to the MS media. After planting, the plates were kept at 4°C for 2 days before being transferred to 22°C under constant illumination with 70% relative humidity for germination and growth. For cold and heat treatments, 11–13 days old seedlings on MS/agar plates were incubated at 0°C (cold) or 37°C (heat) for designated time.

### Construction of *STA1p-LUC* Transgenic Plants

The 1475 bp DNA fragment (-1475 ∼ 0 upstream from the *STA1* translation start codon) of the *STA1* promoter was obtained by polymerase chain reaction using two primers: F9H3.5pBamH1-F; 5′-GTGGATCC ACTTATTGTAGCAATACTTGTTCTTA-3′, and F9H3.5pH3CAM-R; 5′-CCGGT AAGCTTAACCAAACTA TAAAAATCTCT-3′. The promoter fragment was inserted into the BamHI and HindIII site of the binary plant transformation vector pCAMBIA1381Z-LUC, which contains the firefly *LUC* coding sequence (Millar et al., 1992) instead of its original GUS coding sequence. *Arabidopsis thaliana* Columbia *gl1* (Col-*gl1*) plants lacking trichomes were transformed with the resultant *STA1p-LUC* construct using *Agrobacterium tumefaciens* GV1301 strain through floral dipping (Clough and Bent, 1998). Trichome-deficient *Arabidopsis* was selected due to the possible interference of trichomes with the luminescence. The T1 seeds were collected from floral-dipped plants and the transformed T1 seedlings were selected on MS/agar plates containing hygromycin 25 μg/mL (Harrison et al., 2006). Hygromycin tolerant seedlings were transferred into soil pots for continuous growth under normal growth conditions.

### Genetic Analysis of *STA1p-LUC* Transgenic Plants

Copy numbers of *STA1p-LUC* transgene in the selected lines were determined in the progeny derived from either self-crossing or back-crossing. For back-crossing, the selected *STA1p-LUC* plants were back-crossed to the wild-type Col*-gl1* plants and the resultant F1 plants were allowed to self-pollinate. The F2 progeny from either self-crossing or back-crossing were scored for segregation by their luminescence in response to low temperature (0°C for 48 h) and by their hygromycine tolerance on MS/agar plates containing hygromycin 25 μg/mL (Harrison et al., 2006).

### TAIL PCR and Confirmation PCR

For TAIL PCR, three T-DNA specific primers and arbitrary degenerate primers were designed as follows: LB1, 5′-TCCGA GGGCAAAGAAATAGA-3′; LB2, 5′-TTCCTATAGGGTTTCG CTCA-3′; LB3, 5′-TTCTAATTCCTAAAACCAAAATCCA-3′ and DEG1, 5′-WGCNAGTNAGWANAA G-3′; DEG2, 5′-AWGCANGNCWGANATA-3′ (W = A or T; N = A, C, G, or T). The first, second, and third round of tail PCR reactions were carried out as described previously (Liu et al., 1995). For TAIL PCR result confirmation, each PCR was conducted with a primer combination of LB1/2/3, At3g23165-F (5′-CCGGAGGGAATGGAAAATAA-3′) and K14B15-24.42K-R (5′-GGGTCAAACTTGTTTTTCTCG-3′).

### Mutagenesis and Mutant Isolation

Approximately 10,000 seeds from the selected *STA1p-LUC* homozygous line were imbibed overnight in water at 4°C and then soaked in the 0.35% ethyl methanesulfonate (EMS) solution. The seed tube was placed on a rotary shaker set at 30 RPM for 13 h. Treated seeds were rinsed extensively (13 times, 30 min each) in autoclaved water to remove residual EMS. The resulting M1 seeds were sown and grown to set M2 seeds. Approximately 30,000 M2 seedlings from the 326 pools of 20 plants were screened. For imaging screening, the M2 seeds were surface-sterilized and planted individually on MS plates. After the cold and heat treatments, the plates were sprayed immediately with 1 mM luciferin and placed under the luminescence imaging charge-coupled device (CCD) camera (Roper Scientific, US). Luminescence images from M2 seedlings were collected and putative mutants with altered *STA1p-LUC* luminescence were transferred to grow in soil and the resultant M3 seeds were used for further mutant confirmation.

### Measurement of *STA1p-LUC* Luminescence Intensity and Gene Expression

*STA1p-LUC* luminescence was imaged with the lumazone luminescence imaging system (Roper Scientific, US) and luminescence intensity was quantified with the WinView software provided by the camera manufacturer (Chinnusamy et al., 2002). An equal number of pixels of each seedling were selected and total intensity was obtained from a seedling. More than 20 seedlings were quantified for statistical analysis. For gene expression analysis, total RNA was isolated with RNeasy kit (Qiagen, Germany) from 11 to 13 days old seedlings on MS/agar plates with or without stress treatment. Reverse transcription (RT)-PCR was carried out with One-step RT-PCR kit (Qiagen, Germany) for each gene with the following primers; *STA1* (STA1CDS-F2, 5′-CAAGAGTCTGA CCCAGTCGAA-3′; STA1CDS-R2, 5′-AGCCAGAGAACCTCA GCTTG-3′); At3g23165 (At3g23165-F, 5′-CCGGAGGGAATGG AAAATAA-3′; At3g23165-R, 5′-TGTGTTCTTGGTTGGAACT GA-3′); At3g23167 (At3g23167-F, 5′-GCAATCAAACATGCAA TCACA-3′; At3g23167-R, 5′-GCAAAAATGGCATGCAAAC-3′) and Protein phosphatase 2A (PP2A, At1g13320) (PP2A361-F, 5′-GCGTACATCAGGAAATTCGTC-3′; PP2A361-R, 5′-GCGT GTGCGTTATATGGTTG-3′).

## Results

### Construction and Selection of the *STA1p-LUC Arabidopsis*

The 1475 bp of DNA sequence upstream from the *STA1* translation initiation codon was fused to the firefly luciferase coding sequence (*STA1p-LUC*) and used for *Agrobacterium*-mediated *Arabidopsis* transformation to generate T1 seeds for *STA1p-LUC* lines. This *STA1* upstream area was used as a promoter sequence because successful molecular complementation of *sta1-1* by this promoter-driven *STA1* coding sequence demonstrated functional activity of this region (Lee et al., 2006).

**FIGURE 1:**
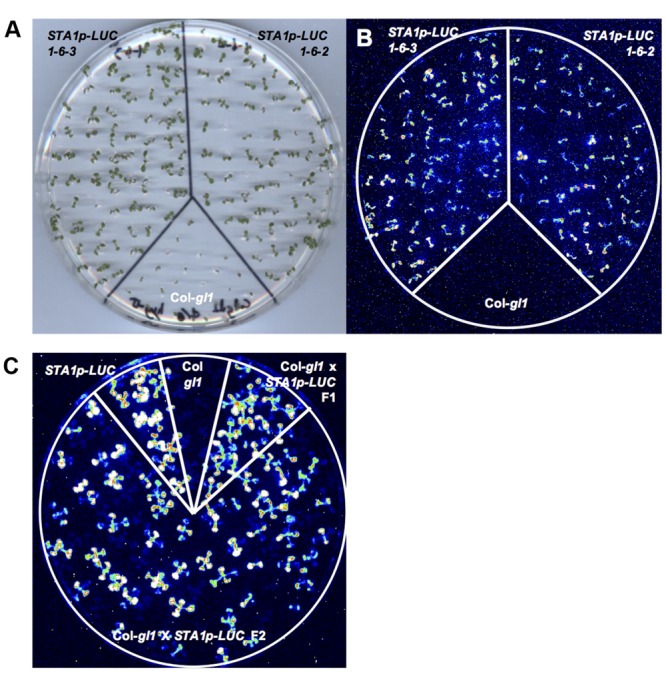
**Luminescence analysis of *STA1p-LUC* T4 lines. (A)** Plate containing 11 day-old T4 seedlings, used for luminescence image **(B)**. **(B)** Luminescence image of T4 seedlings after cold stress treatment (0°C for 2 days). **(C)** *STA1p-LUC* luminescence in progeny lines derived from Col-*gl1* x *STA1p-LUC* cross. Luminescence image of 13 days old seedlings was taken after cold stress treatment (0°C for 2 days). Col-*gl1* was a negative control.

The T1 seeds were planted on MS agar media with hygromycin, and hygromycin-tolerant T1 transgenic plants were selected and transplanted to soil. These T1 plants were likely hemizygous for the *STA1p-LUC* transgene and the hygromycin resistance gene on the transformation vector. The T2 progeny from the hygromycin-tolerant T1 plants were further analyzed for the zygosity of functional *STA1p-LUC* transgene and its cosegregation with the hygromycin resistance gene. Eleven day-old T2 seedlings on plates were cold-treated at 0°C for 2 days, and two T2 lines (#1 and #6) showing a 3:1 segregation ratio of luminescence presence to absence were selected (**Table 1**). This 3:1 segregation suggested a single insertion of *STA1p-LUC* in these lines. Hygromycin resistance was also tested with these two lines (#1 and #6) and resulted in a 3:1 segregation of hygromycin resistance to non-resistance in only line #1, indicating a single insertion of the resistance gene in this line (**Table 1**). PCR analysis confirmed that all luminescent seedlings of line #1 contained the hygromycin resistance gene and all hygromycin-resistant seedlings of line #1 contained the luciferase transgene (data not shown) which suggested a cosegregation of *STA1p-LUC* and the hygromycin resistance gene. The luminescence and hygromycin resistance segregation was further examined in the T3 and T4 generations derived from line #1. In these generations, we expected to identify lines homozygous for *STA1p-LUC*. Lines #1–2 and #1–4 showed an approximate 3:1 segregation of hygromycin resistance to non-resistance, while nearly all seedlings of #1–1 and #1–6 were tolerant to hygromycin, indicating that the lines of #1–1 and #1–6 were homozygotes for hygromycin resistance and likely also for *STA1p-LUC*. Indeed, the seedlings of #1–1 and #1–6 also emitted luminescence from almost all seedlings, whereas the seedlings of #1–2 and #1–4 produced a 3:1 ratio of luminescence presence to absence. The two lines of T4 generation derived from #1–6 (#1–6–2 and #1–6–3) showed near-perfect luminescence and hygromycin resistance (**Figures 1A,B**). In addition, we could not detect meaningful segregation of luminescence or hygromycin resistance in the progeny seedlings of these lines in the next generation (T5; Supplementary Table S1). Among the progeny seedlings of these lines, the few seedlings that did not show luminescence and hygromycin resistance appeared to be physiological variations because PCR analysis of the seedlings revealed specific bands for both luciferase coding sequence and hygromycin resistance gene (data not shown). Taken together, genetic analysis through several generations demonstrated that the progeny lines were homozygous for *STA1p-LUC* and hygromycin resistance (i.e., #1–1, #1–6, #1–6–2, and #1–6–3).

**Table 1:** Genetic analysis of *STA1p-LUC* lines.

Lines		Luminescence					Hygromycin				
		Present	Absent	Ratio^∗^	χ^2^	*p*-value^†^	Resistant	Sensitive	Ratio^∗^	χ^2^	*p*-value^†^
T2	1	76	25	3.04:1	0.003	0.95	61	21	2.90:1	0.016	0.90
	6	57	17	3.35:1	0.162	0.69	24	48	0.50:1	66.667	<0.0001
T3	1–1	86	4	100:4.65			53	0	100:0		
	1–2	68	22	3.09:1			47	15	3.13:1		
	1–4	70	25	2.80:1			33	13	2.54:1		
	1–6	88	0	100:0			54	0	100:0		
T4	1–6–2	223	0	100:0			232	0	100:0		
	1–6–3	239	2	100:0.84			246	2	100:0.81		
Col*-gl1* × 1–6–2 F2		68	24	2.83:1	0.058	0.81	76	25	3.04:1	0.003	0.95
1–6–2 × Col*-gl1* F2		81	32	2.53:1	0.664	0.42	92	33	2.79:1	0.131	0.72
Col*-gl1* × 1–6–3 F2		71	22	3.23:1	0.090	0.76	92	30	3.07:1	0.011	0.92
1–6–3 × Col*-gl1* F2		76	26	2.92:1	0.013	0.91	110	37	2.97:1	0.002	0.96

*^∗^Ratio indicates the ratio of plants with luminescence presence to absence.*

*^∗∗^Ratio indicates the ratio of plants with hygromycin resistance to sensitivity.*

*†The *p*-value is the probability calculated from the χ^*2*^value and the *p*-values greater than the conventional threshold of 0.05 indicate that the data fit a 3:1 ratio.*

The single insertion of T-DNA was genetically reconfirmed by crossing the *STA1p-LUC* hymozygote lines to the original background line (Col-*gl1*). F2 generation from the cross between *STA1p-LUC* lines (#1–6–2 or #1–6–3) and Col-*gl1* showed an approximate 3:1 segregation in both luminescence to no-luminescence and hygromycin-resistance to non-resistance (**Table 1** and **Figure 1C**), which confirmed that both #1–6–2 and #1–6–3 lines contained a single homozygous insertion of *STA1p-LUC* transgene in each genome. For further analysis, we used #1–6–2 line as our *STA1p-LUC* line.

### Identification of the *STA1p-LUC* Insertion Position

**FIGURE 2:**
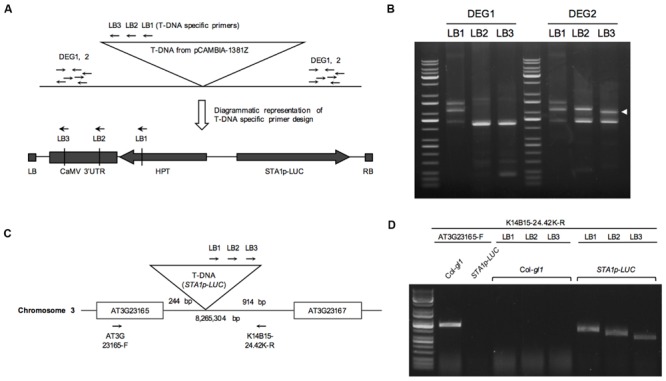
**Thermal asymmetric interlaced (TAIL) PCR of *STA1p-LUC*. (A)** Schematic diagram of TAIL PCR to show primer binding sites. **(B)** TAIL PCR results of *STA1p-LUC*. White arrowhead indicates a third round PCR product showing a TAIL PCR-typical shifted band pattern along with the first and second round PCR products in gel, which is likely a specific PCR band from the T-DNA flanking region. PCR primer pairs were labeled on the top. **(C)** Schematic diagram of positions of T-DNA insertion and primers designed for confirmation of TAIL PCR results. T-DNA insertion occurred at 8,265,304th bp of chromosome 3, and 244 bp and 914 bp away from AT3G23165 and AT3G23167, respectively. **(D)** TAIL PCR confirmation. Primer pairs used are shown on the lines and plant genomic DNA used are on top of the gel picture.

**FIGURE 3:**
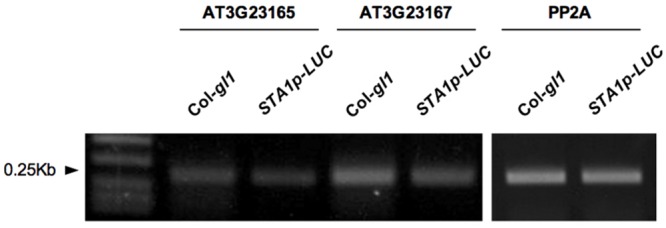
**Semi-quantitative RT-PCR for *STA1p-LUC* neighboring genes expression.** Expression of two *STA1p-LUC* T-DNA adjacent genes (AT3G23165 and AT3G23167) was analyzed by semi-quantitative RT-PCR. PP2A (At1g13320) was used as control.

Thermal Asymmetric Interlaced (TAIL) PCR is a simple and powerful method to identify DNA sequence adjacent to known sequences (Liu et al., 1995). In order to locate the position of the *STA1p-LUC* transgene insertion, the flanking region adjacent to the left border of the T-DNA of *STA1p-LUC* was amplified by TAIL PCR. We targeted left-border neighboring DNA because left borders of T-DNA tend to remain intact more frequently than right borders during T-DNA insertion (Weigel et al., 2000). Three nested T-DNA left border specific primers (LB1, LB2, and LB3) with approximately 100 bp distance were used together with arbitrary degenerate primers (DEG1 and DEG2; **Figure 2A**). The resultant PCR products of each round reaction were fractionated in agarose gel, and gel-eluted DNAs from distinct bands were sequenced (**Figure 2B**). Blast search revealed that DNA sequences corresponded to the sequences starting from the 8,265,304th nucleotide on the chromosome 3. The locus was located between At3g23165 and At3g23167, both of which encode a member of a family of small, secreted cysteine-rich proteins similar in sequence to a pollen coat protein. Predicted T-DNA insertion was verified by PCR using two pairs of primers specific for T-DNA left border and the T-DNA-flanking *Arabidopsis* DNA sequences. (LB1, LB2, or LB3 and K14B15-24.42-R; At3g23165-F and K14B15-24.42K-R; **Figure 2C**). As shown in **Figure 2D**, the primer pair spanning T-DNA left border and the left-border flanking region showed distinct PCR bands, the sizes of which were gradually reduced according to the expected size reduction for the LB primers (LB1, LB2, and LB3). Also, the primers located on left and right T-DNA flanking region (At3G23165-F and K14B15-24.42K-R) produced specific PCR bands in control DNA (Col-*gl1*) but not in *STA1p-LUC* line (**Figure 2D**). These results confirmed that the *STA1p-LUC* transgene was homozygously inserted between At3g23165 and At3g21167. The insertion position of the *STA1p-LUC* trangene was 244 bp away from the translation start of Atg323165 and 914 bp away from the 3′ end of At3g23167 coding sequence (**Figure 2C**). Additionally, we tested whether the transgene altered the expression of adjacent genes (AT3G23165 and AT3G23167) using semi-quantitative RT-PCR and found that no meaningful expression alteration was caused by the *STA1p-LUC* transgene insertion (**Figure 3**). Thus, we concluded that the *STA1p-LUC* line was successfully generated without disrupting neighboring genes.

### Expression of *STA1p-LUC* under Temperature Stresses

Despite its “housekeeping” functions, *STA1* is a temperature stress-inducible gene (Lee et al., 2006). To test whether the expression patterns of *STA1p-LUC* are consistent with those of the endogenous *STA1* expression, we measured the *STA1p-LUC* luminescence intensity and the endogenous *STA1* expression levels in *STA1p-LUC* plants under heat and cold stress conditions.

**FIGURE 4:**
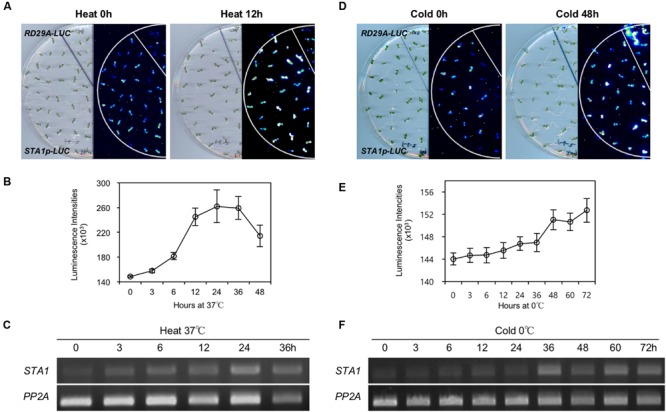
***STA1p-LUC* luminescence and endogenous *STA1* expression in *STA1p-LUC* seedlings after heat or cold treatment. (A)** and **(D)** Plate and corresponding luminescence images of *STA1p-LUC* seedlings before and after heat or cold treatment. RD29A-LUC was used as a positive control and did not show the heat-induction of luminescence. **(B,E)** Quantification of luminescence intensities of *STA1p-LUC*. Luminescence intensities from at least 20 seedlings were measured and averaged. Error bars represent standard deviation. **(C,F)** Semi-quantitative RT-PCR for the endogenous *STA1* expression. PP2A (At1g13320) was used as a loading control.

Under heat conditions, *STA1p-LUC* intensity increased until 24 h and then decreased gradually (**Figures 4A,B**). The expression patterns of endogenous *STA1* gene under heat stress were similar to those of *STA1p-LUC* (**Figure 4C**). Under cold stress, the *STA1p-LUC* expression was gradually induced until 72 h (**Figure 4E**). Similarly, the endogenous *STA1* expression was upregulated with a slight fluctuation under cold stress (**Figure 4F**). However, cold-induction of both *STA1p-LUC* and the endogenous *STA1* was not as high as heat induction (**Figure 4**). Taken together, these results demonstrated that *STA1p-LUC* expression of the *STA1p-LUC* line reliably reflected endogenous *STA1* expression.

### Isolation of Mutants with Altered *STA1p-LUC* Expression under Temperature Stresses

**FIGURE 5:**
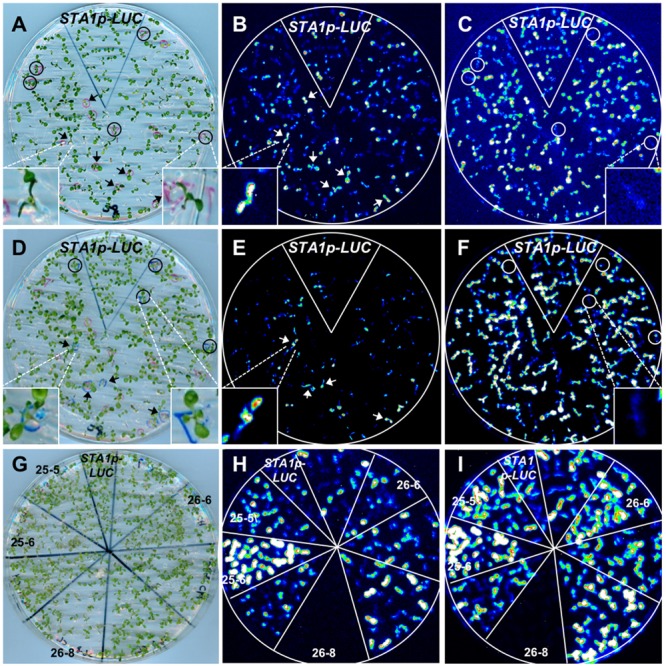
***STA1p-LUC* deregulated mutant screening. (A)** Plate containing 11 day-old M2 seedlings for **(B)** and **(C)**. **(B)** and **(C)** Luminescence image taken after cold stress treatment (0°C for 36 h). **(D)** Plate containing 13 day-old seedlings for **(E)** and **(F)**. The same, cold-treated plate **(A)** was used for heat stress after 1 day adaptation at normal temperature. **(E)** and **(F)** Luminescence image taken after heat treatment (37°C for 15 h). In comparison with *STA1p-LUC*, putative mutants with higher luminescence were marked with arrows and those with lower luminescence were marked with circles. Insets in **(A–F)** are the representative images of either seedlings or luminescence. Left inset in **(D)** shows a seedling with one leaf slightly overlapped by a leaf of a nearby seedling. **(G)** Plate containing 11 day-old putative mutant seedlings (M3 generation) for confirmation **(H,I)**. **(H)** and **(I)** Luminescence images taken after cold stress **(H)** and heat stress **(I)**. The confirmed mutants without segregation were labeled with mutant numbers and the results showed two *tis* (25-5 and 25-6), one *his* (26-6), and one *trs* (26-8).

As the *STA1p-LUC* line faithfully reflected endogenous *STA1* expression, we decided to use the *STA1p-LUC* line to isolate the genetic loci for the regulators of *STA1* expression. Thus, *STA1p-LUC* seeds were chemically mutagenized with EMS, and the resulting plants (M1 generation) were allowed to self-pollinate. The following M2 generation was screened to isolate the mutants with altered *STA1p-LUC* expression under cold or heat stress using an luminescence imaging system (**Figures 5A–F**; Chinnusamy et al., 2002). Putative M2 mutant individuals emitting altered *STA1p-LUC* luminescence under temperature stresses were transferred to soil and the next generation (M3) seeds from each M2 mutant were individually harvested. After the first screening with approximately 30,000 M2 seedlings from the 326 pools of 20 plants, 528 putative mutants were selected. Among these, two hundred lines survived and set seeds (M3).

**FIGURE 6:**
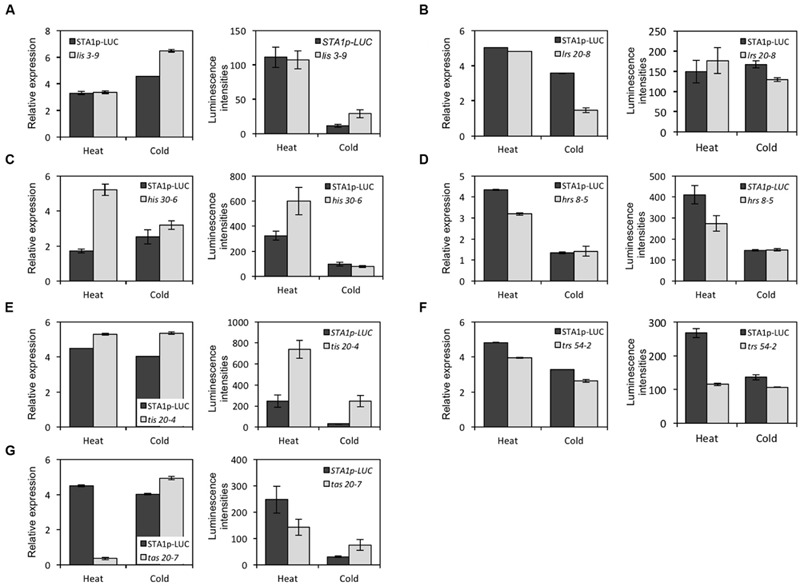
**The endogenous *STA1* expression and the *STA1p-LUC* luminescence in confirmed mutants in response to heat and cold stresses.** Graphs in the left column in each letter-labeled set are the endogenous *STA1* expression levels by quantitative RT-PCR (three replicates; bar = standard deviation) and graphs in the right column are *STA1p-LUC* luminescence intensities. 20–25 seedlings were used to measure expression levels. Error bars represent standard deviation. **(A)** *lis* (*low temperature increased STA1p-LUC expression*). **(B)** *lrs* (*low temperature reduced STA1p-LUC expression*). **(C)** *his* (*high temperature increased STA1p-LUC expression*). **(D)** *hrs* (*high temperature reduced STA1p-LUC expression*). **(E)** *tis* (*temperature increased STA1p-LUC expression*). **(F)** *trs* (*temperature reduced STA1p-LUC expression*). **(G)** *tas* (*temperature altered STA1p-LUC expression*).

In the M3 generation, confirmation imaging was conducted to validate the putative mutants (**Figures 5G–I**). M3 putative mutant lines were confirmed as mutants when all siblings of the lines showed the altered *STA1p-LUC* expression. In addition, we tested the expression levels of endogenous *STA1* in these confirmed lines (M3) and identified 23 *STA1p-LUC* deregulated mutants having the same alterations of endogenous *STA1* expression under temperature stresses (**Figure 6**). These mutants were then classified on the basis of luminescence intensities under either low or high temperatures (**Table 2**).

**Table 2:** Categorization of *STA1p-LUC* mutants with altered luminescence.

Mutant Class	Number
*lis* (low temperature increased *STA1p-LUC* expression)	2
*lrs* (low temperature reduced *STA1p-LUC* expression)	1
*his* (high temperature increased *STA1p-LUC* expression)	6
*hrs* (high temperature reduced *STA1p-LUC* expression)	1
*tis* (temperature increased *STA1p-LUC* expression)	7
*trs* (temperature reduced *STA1p-LUC* expression)	4
*tas* (temperature altered *STA1p-LUC* expression)	2^∗^

*^∗^High *STA1p-LUC* luminescence by cold and low *STA1p-LUC* luminescence by heat.*

The classified mutants were named as follows: *lis* (Low-temperature Increased *STA1* promoter-driven luciferase expression), *lrs* (Low temperature Reduced *STA1* promoter-driven luciferase expression), *his* (High temperature Increased *STA1* promoter-driven luciferase expression), *hrs* (High temperature Reduced *STA1* promoter-driven luciferase expression), *tas* (Temperature Altered *STA1* promoter-driven luciferase expression), *tis* (Temperature Increased *STA1* promoter-driven luciferase expression), *trs* (Temperature Reduced *STA1* promoter-driven luciferase expression).

Some mutants showed alteration of *STA1p-LUC* expression by both cold and heat stresses (*tis, trs*, and *tas*), while others showed the deregulation of *STA1p-LUC* specifically by either cold or heat (*lis, lrs, his*, and *hrs*). These observations suggested the existence of common genetic regulators as well as heat or cold stress-specific regulators for *STA1* expression. Interestingly, *tas* mutants were identified due to their opposite-directional deregulation of *STA1p-LUC* depending on the kind of thermal stress. Two mutants showed higher luminescence intensities of *STA1p-LUC* under cold and lower *STA1p-LUC* expression under heat in comparison to its background line, and these mutants also displayed similar alterations of endogenous *STA1* expression (i.e., increased *STA1* expression in cold and reduced *STA1* expression in heat). This result suggests that these *tas* mutants may be defective in important switch genes directing thermal-stress signaling pathways for *STA1* expression.

## Discussion

The *Arabidopsis STA1* gene is present as a single copy in the genome and encodes a pre-mRNA splicing factor with high homology to the budding yeast pre-mRNA splicing factors, Prp6p and the human U5 snRNP-associated 102-kDa protein, PRPF6. *Arabidopsis* is known to have a total of 14 U5 snRNP specific proteins including *STA1* (Wang and Brendel, 2004). Among them, our database search revealed that only *STA1* and one *Brr2* homolog (At2g42270) were highly induced by thermal stresses. Although the *sta1-1* mutants have shown temperature stress-hypersensitivity, the mutant phenotypes of the temperature-induced *Brr2* homolog (At2g42270) have not been reported so far. Thus, *STA1* provides a good opportunity to study how this seemingly “housekeeping” gene is specifically required under unfavorable temperatures, and how thermal stress signaling pathways are interconnected. One possible explanation for thermal stress-specific phenotypes of *sta1-1* is that *sta1-1* is a temperature sensitive allele, but *sta1-1* has shown developmental defects under normal conditions (Lee et al., 2006). Thus, *sta1-1* mutation does not seem to be a temperature sensitive allele which should otherwise display normal phenotypes under normal conditions. Another explanation is that *STA1* itself is specifically required under unfavorable temperatures; thus, thermal stress-induction of *STA1* might be correlated with its function in cold or heat stress.

As a first step toward understanding *STA1* induction and specificity under cold and heat, we generated the *STA1p-LUC* bioluminescent *Arabidopsis* plant. The faithfulness of our *STA1p-LUC* line system was verified by comparing the *STA1p-LUC* expression patterns with endogenous *STA1* expressions. In addition, the homozygous single-copy *STA1p-LUC* insertion was confirmed not to interfere with the insertion-neighboring gene expression (**Figure 3**). In some cases, foreign gene insertion with 35S promoters resulted in altered expression of the genes adjacent to the insertion (Yoo et al., 2005; Zheng et al., 2007; Singer et al., 2010, 2011).

Several kinds of bioluminescent *Arabidopsis* plants have been developed to study gene expression regulation (Ishitani et al., 1997; Chinnusamy et al., 2002; Yao et al., 2014). In our study, the mutagenesis of *STA1p-LUC* lines and mutant screening identified many luminescence-altered mutants under cold and heat stresses. While some mutants altered *STA1p-LUC* expression under only cold or heat stress conditions, others affected *STA1p-LUC* expression under both. These results suggested the existence of independent signaling pathways for each stress, and also the presence of diverse cross-talks and shared signaling pathways between cold and heat stress responses. Heat shock transcription factors (HSF) and heat shock proteins (HSP) are induced by multiple stresses and are thought to be an interacting point between heat and non-heat stress responses (Swindell et al., 2007). Swindell et al. (2007) suggested that HSF and HSP induction by multiple stresses might be mediated by secondary oxidative stress. Interestingly, *STA1* was strongly induced primarily by temperature stresses but remained almost unaffected by other stresses, implicating that *STA1* induction by thermal stresses might not be mediated by oxidative stress. Taken together, our *STA1p-LUC* mutants might only represent the genetic factors involved in more direct thermal-stress signaling pathways for *STA1* regulation rather than indirect oxidative pathways.

Multiple cross-talks and specific signaling pathways among the stress signaling pathways are not uncommon. For example, *Arabidopsis* full-length cDNA microarray analyses revealed that more than half of drought-induced genes were also induced by high salt stress, implicating the presence of cross-talks between salt and drought stress signaling for gene induction (Seki et al., 2002). However, to our knowledge, direct comparisons of whole genome expression profiles between heat and cold stresses have not been carried out. This might be because of the assumption that plants may not undergo such dramatic temperature changes (e.g., -5 to 35°C). Nevertheless, public genomics data suggested that there are some common gene sets that are regulated by both cold and heat. Indeed, the comparative metabolomics revealed that many metabolites were commonly altered by heat and cold; some were changed only by each specific stress (Kaplan et al., 2004). These findings suggest that plants have mechanisms which use common and specific signaling networks for such dramatic temperature changes. Changes in membrane fluidity, internal Ca^2+^ levels and protein unfolding levels are among the common cellular responses that happen early during both heat and cold stresses (Sung et al., 2003; Ruelland and Zachowski, 2010). Therefore, some of our *STA1p-LUC* mutants deregulated by both cold and heat might have defects in these interconnection points. Interestingly, we isolated two *tas* mutants which showed *STA1* up-regulation by cold, but down-regulation by heat. These genetic loci may represent the decision-making branching points in the signaling pathways. Cloning of genes responsible for *STA1p-LUC* deregulation in our mutants will help in identifying common and specific thermal-stress signaling pathways for *STA1* regulation and shed light on the regulation of temperature stress-induction of this “housekeeping” gene.

## Conclusion

In this study, we have generated a bioluminescent *Arabidopsis* plant harboring a single copy of a *STA1* promoter-driven firefly luciferase (*STA1p-LUC*), which faithfully reflected the gene expression patterns of endogenous *STA1* under cold and heat stresses. Mutagenesis was performed using *STA1p-LUC* as a background line, and mutants showing deregulation of the transgenic *STA1p-LUC* and endogenous *STA1* gene under cold and heat stresses were successfully isolated. The isolated mutants suggested the existence of genetic loci for stress-specific and shared signaling components of *STA1* regulation. These genetic loci may also include important genetic switches to determine the direction of cold and heat signaling pathways.

## Author Contributions

B-hL conceived and designed the research; S-iY, CC, and J-HH performed the experiments; S-iY, J-HH and B-hL discussed the results and wrote the paper.

## Conflict of Interest Statement

The authors declare that the research was conducted in the absence of any commercial or financial relationships that could be construed as a potential conflict of interest.
