# Supplementary material for: Genetic Screening for Arabidopsis Mutants Defective in STA1 Regulation under Thermal Stress Implicates the Existence of Regulators of Its Specific Expression, and the Genetic Interactions in the Stress Signaling Pathways
Source: Front Plant Sci. 2016 May 10;7:618. doi: 10.3389/fpls.2016.00618 (PMC4861721; doi:10.3389/fpls.2016.00618)
Supplement: Supplementary file 1 [file Table_1.PDF]

Supplementary Table 1. Genetic analysis of *STA1p-LUC* lines in T5 generation

Lines		Luminescence			Hygromycin		
		Present	Absent	Percentage (%)*	Resistant	Sensitive	Percentage (%)*
T5	1-6-2-2	125	12	91.2	134	3	97.8
	1-6-2-4	87	10	89.7	95	2	97.9
	1-6-3-1	101	3	97.1	104	0	100
	1-6-3-4	104	7	93.7	111	0	100
	1-6-3-6	93	7	93.0	96	4	96.0
	1-6-3-14	100	3	97.1	102	1	99.0
	1-6-3-15	86	4	95.6	89	1	98.9
	1-6-3-16	88	1	98.9	89	0	100
	1-6-3-17	104	8	92.9	110	2	98.2
	1-6-3-20	109	7	94.0	115	1	99.1

* Percentage means the dominant phenotype (luminescence or hygromycin-resistance) percentage in total number of seedlings analyzed.
